# Continuous blood pressure monitoring via hemodynamic parameter and pulse transit time derived from capacitive sensing pads

**DOI:** 10.1088/1361-6579/ae575a

**Published:** 2026-04-08

**Authors:** Yu-Jen Cheng, Edward Kim, Jin-Oh Hahn, Jae-Hyun Chung, Younghoon Kwon

**Affiliations:** 1 Mechanical Engineering Department, University of Washington, Seattle, WA 98195, United States of America; 2 Division of Cardiology, University of Washington, Seattle, WA 98195, United States of America; 3 Mechanical Engineering Department, University of Maryland, College Park, MD 20742, United States of America

**Keywords:** continuous blood pressure monitoring, nocturnal hypertension, sensing pad

## Abstract

*Objective.* Continuous blood pressure (BP) monitoring is crucial for detecting nocturnal hypertension and acute hemodynamic changes. Conventional cuff-based methods disrupt sleep and miss transient BP fluctuations from sleep-related events or instability. Cuffless methods, such as pulse transit time (PTT), offer potential but often struggle to reliably track acute BP fluctuations due to complex and nonlinear hemodynamics. *Approach.* We developed a capacitive sensing pad system incorporating PTT with additional hemodynamic features for unobtrusive, continuous BP monitoring in supine subjects. The pad contains ultra-sensitive single-electrode capacitive (SEC) sensors made from carbon nanotube composites. In passive contact, SEC sensor on the back of the chest captures intrathoracic blood volume changes through deep tissue permittivity and ballistocardiography, while another under the leg detects arterial pulse-induced tissue vibrations. PTT is derived from the temporal delay between chest and leg signals. A neural network model incorporates PTT and intrathoracic blood volume features to improve BP estimation. *Main results.* In human trials (*N* = 30) with arm-cuff reference, the system showed strong correlation (*r* ⩾ 0.94), with a mean error (ME) ⩽ 0.1 mmHg and standard deviation (SD) ⩽ 5.6 mmHg. In a subset (*N* = 8) with continuous finger-cuff reference, it maintained strong correlation (*r* ⩾ 0.89), with a ME ⩽ 0.2 mmHg and SD ⩽ 7.8 mmHg. *Significance.* These results suggest the possibility of bed-based unobtrusive and continuous BP monitoring in the supine position.

## 1. Introduction

High blood pressure (BP) is a leading risk factor for cardiovascular disease, affecting more than one-third of adults worldwide (Mills *et al* 2020). Continuous BP monitoring can be useful in certain clinical contexts including detection of nocturnal hypertension, a clinical phenotype strongly associated with adverse cardiovascular disease and mortality (Hansen *et al* 2011, Cuspidi *et al* 2016, Kwon *et al* 2020). It also provides critical insights into hemodynamic stability in supine or bedridden patients, such as those in postoperative recovery or critical care (Futier *et al* 2017). Despite its clinical importance, continuous BP monitoring during rest or sleep remains challenging (Davies *et al* 1994). Standard ambulatory BP monitoring uses oscillometric cuff inflations every 30–60 min, which is disruptive (Gaffey *et al* 2021). More critically, intermittent measurements fail to capture episodic BP fluctuation triggered by sleep-related events such as arousals and obstructive sleep apnea, (Somers *et al* 1995, Kwon *et al* 2022a) as well as acute hemodynamic instability in hospitalized patients (Wesselink *et al* 2018).

Continuous, unobtrusive BP monitoring is essential for detecting these BP fluctuations during sleep or rest, Sato *et al* (2025). However, current noninvasive technologies often involve trade-offs between accuracy, comfort, and practicality. Cuff-based systems, such as volume-clamp and pulse decomposition methods (Imholz *et al* 1993, Kwon *et al* 2022a, Kwon Y *et al* 2022b), continuously apply pressure to the finger and are typically bulky and uncomfortable, hindering the prolonged or nocturnal use.

Cuffless technologies, such as photoplethysmography (PPG)-based wearable devices, estimate BP from peripheral pulse waveforms (Mukkamala *et al* 2022). While convenient, they are highly sensitive to contact pressure, motion artifacts, and intra- and inter-subject vascular variability, requiring frequent cuff-based calibration. Pulse transit time (PTT), the time a pulse wave takes to travel between two arterial sites, is inversely related to BP and widely used for BP estimation (Mukkamala *et al* 2015, Ding and Zhang 2019, Yousefian *et al* 2019). However, due to the nonlinear and complex nature of hemodynamics, PTT alone may not reliably track acute BP fluctuations, as it is influenced by factors beyond arterial pressure, including cardiac output and vasomotor tone (Ding and Zhang 2019). Many systems use pulse arrival time as a surrogate for PTT, derived from electrocardiography (ECG) and PPG, for easier setup and installation (Gesche *et al* 2012, Patzak *et al* 2015). However, the inclusion of the pre-ejection period introduces additional variability and further reduces accuracy (Nyvad *et al* 2021).

These limitations highlight the need for a continuous, unobtrusive BP monitoring method, particularly during sleep and in acute care settings. This study presents a capacitive sensing pad system for continuous BP monitoring in supine subjects. The pad integrates pressure-sensitive single-electrode capacitive (SEC) sensors made from carbon nanotube composites to detect intrathoracic blood volume changes and ballistocardiography (BCG) at the back of the chest, and pulse vibrations at the leg. PTT was derived from these signals, and a neural network estimated BP using PTT, intrathoracic blood volume, and other physiological features. The contribution of intrathoracic blood volume was evaluated, and the system was validated against cuff-based measurements.

## 2. Methods

### 2.1. Mechanism for continuous BP monitoring via capacitive sensing pads

**Figure 1.**
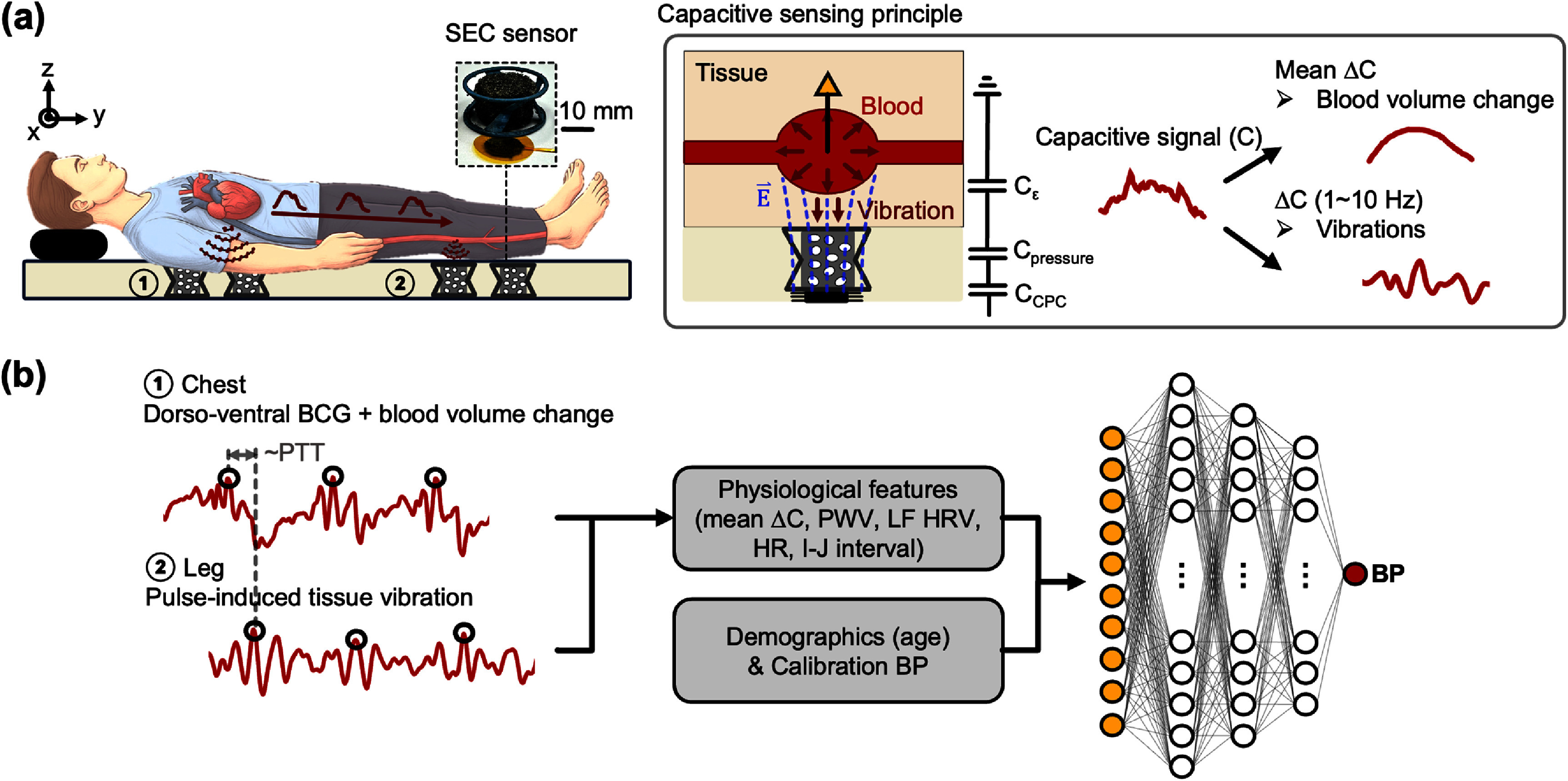
(a) Schematic illustrations of the capacitive sensing pads embedded with SEC sensors, sensing mechanism and equivalent circuit model. The capacitive signal is modeled as three serially connected components: *C*_CPC_ (CPC electrode), *C*_pressure_ (pressure), and *C_ϵ_* (blood volume). Blood acts as a floating ground. As blood volume changes, this ground shifts, modulating capacitance. Frequency-based processing allows differentiation between blood volume changes (<0.5 Hz) and cardiac-induced vibrations (1–10 Hz). (b) Representative SEC signals from the chest and leg, with an overview of the BP estimation pipeline. Physiological features are extracted from preprocessed SEC signals, then combined with demographic information and calibration BP to train a neural network.

Capacitive sensing pads embedded with previously developed SEC sensors were positioned under the chest and near the popliteal region in supine subjects to calculate PTT (figure 1(a)) (Cheng *et al* 2024). We tested the hypothesis that BP estimation would be enhanced by incorporating mean capacitance variation (mean Δ*C*), which detected intrathoracic blood volume changes.

Each SEC sensor consisted of a carbon nanotube paper composite (CPC) electrode and a pressure-sensitive foam (figure 1(a)). Previous studies have shown that the SEC sensor can sense deep tissue signals, enabling the detection of capacitance changes associated with intrathoracic blood volume variations and cardiac-induced vibrations (Cheng *et al* 2024, 2025). In our previous animal hemorrhage study, mean Δ*C* correlated with mean arterial pressure (MAP; cross-correlation = 0.76) as blood volume decreased. MAP served as a surrogate for blood volume, reflecting the product of cardiac output and vascular resistance (Cheng *et al* 2025). In brief, without an electrical ground, the SEC sensor’s electric field extends deeply into the tissue and ultimately terminates in the blood network. Given the high conductivity of blood, the blood network effectively behaves like a floating ground. Changes in blood volume altered the field distribution and modulated capacitance, while the high-aspect ratio CPC electrode enhanced this modulation by strengthening the electric field. The pressure-sensitive foam (0.2 Pa sensitivity) enabled passive and nonwearable detection of BCG and pulsation.

The SEC signal was modeled as three serially connected components to elucidate the underlying signal sources (figure 1(a)): *C*_CPC_ (CPC electrode capacitance), *C*_pressure_ (pressure-induced capacitance), and *C_ϵ_* (blood volume–dependent capacitance). The chest sensor detected intrathoracic blood volume changes and dorso-ventral BCG (figure 1(b)). The leg sensor captured pulse-induced tissue vibrations associated with major leg arteries, with dominant peaks aligned to the systolic popliteal pulse wave, as confirmed by reference measurements (figure S1). BCG exhibited dominant frequency components between 1–10 Hz (Kim *et al* 2018), whereas intrathoracic blood volume changes occurred at lower frequencies (<0.5 Hz), primarily reflecting respiratory modulation and global stroke volume variation (Mischi *et al* 2009). Accordingly, frequency analysis separated these components (figure 1(a)): mean Δ*C* (<0.5 Hz) represented blood volume changes, while the 1–10 Hz band corresponded to cardiac vibrations analogous to BCG. Chest mean Δ*C* (figure S2) exhibited a larger amplitude than leg mean Δ*C* as it reflects stroke volume (vs peripheral pulse volume). These signals provided the basis for deriving mean Δ*C*, PTT, and BCG-related features, which were subsequently used for BP estimation.

### 2.2. Patients and data collection

The study was approved by the institutional review board (IRB) at University of Washington (UW) (IRB ID: STUDY00019217). Informed written consent was obtained from all participants prior to the research. The study included 30 subjects (aged 18–80 years, mixed gender) with a normotensive to hypertensive ratio of 6–4 (table 1). The classification of normal BP and hypertension was based on the 2025 American Heart Association guidelines (Jones *et al* 2025).

**Table 1.** Baseline characteristics of patients.

Characteristics	Mean (SD) or *N* (%)
Age (years)	41.0 (20.8)
BMI (kg m^−2^)	24.7 (6.6)
Male	20 (66.7%)
Female	10 (33.3%)

Baseline blood pressure	N (%)

Normal: SBP < 120 mmHg and DBP < 80 mmHg	17 (56.7%)
Elevated: SBP 120–129 mmHg and DBP < 80 mmHg	2 (6.7%)
Stage 1 hypertension: SBP 130–139 mmHg or DBP 80–89 mmHg	5 (16.7%)
Stage 2 hypertension: SBP ⩾ 140 mmHg or DBP ⩾ 90 mmHg	6 (20.0%)

Reference intermittent BP was measured using an oscillometric arm cuff (OMRON BP5250). In a subset of 8 subjects, reference continuous BP was obtained using an FDA- and CE-cleared finger cuff monitor (VitalStream, Caretaker Medical, VA, USA). VitalStream employed pulse decomposition analysis to provide beat-to-beat BP measurements (Baruch *et al* 2011, Kwon *et al* 2022b). The finger cuff was worn on the middle finger of the arm opposite the arm cuff to avoid interference from cuff inflation/deflation. Although inter-arm differences might exist, the two reference measurements were analyzed independently.

**Figure 2.**
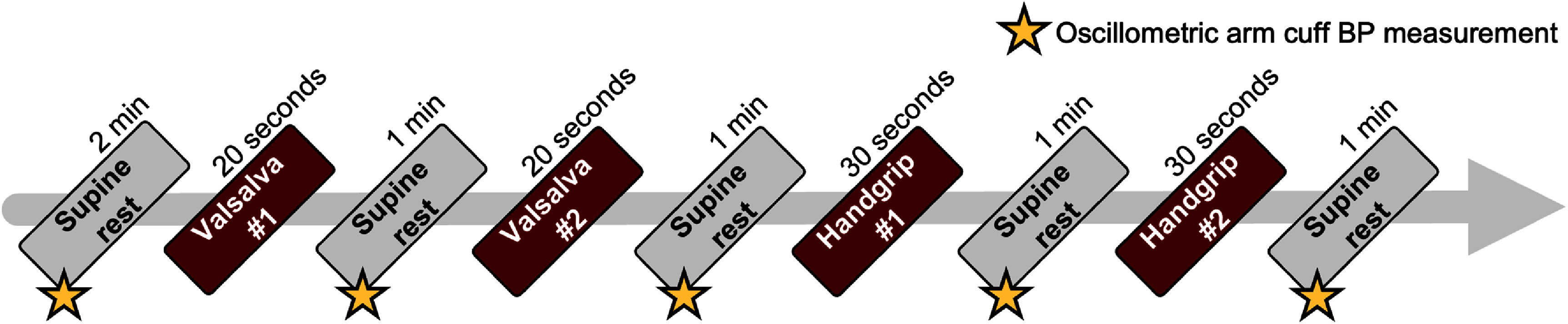
Flowchart of the testing protocol incorporating BP-perturbing maneuvers (Valsalva and sustained isometric handgrip). Intermittent reference BP was measured during supine rest, whereas continuous reference BP was recorded throughout the testing period.

Subjects lay supine on two capacitive sensing pads placed on top of a mattress, one under the posterior thorax and the other under the leg near the popliteal region, with spacing adjusted according to subject’s anthropometric characteristics (figure S3). After a 2 min rest period, baseline BP was measured with the arm cuff for calibration prior to testing (figure 2). To induce BP variability, subjects performed a series of maneuvers while remaining supine. These included two 20 s Valsalva maneuvers and 30 s sustained isometric handgrip exercises, each followed by a 1 min rest. The Valsalva maneuvers were performed by forceful exhalation against a closed airway while keeping the mouth and nose closed (Porth *et al* 1984). The isometric handgrip involved squeezing a rolled towel continuously for 30 s. Intermittent reference BP was measured during post-maneuver supine rest to avoid short-term hemodynamic variability during the maneuver, whereas continuous reference BP was recorded throughout the entire protocol.

### 2.3. Signal processing and feature extraction

**Figure 3.**
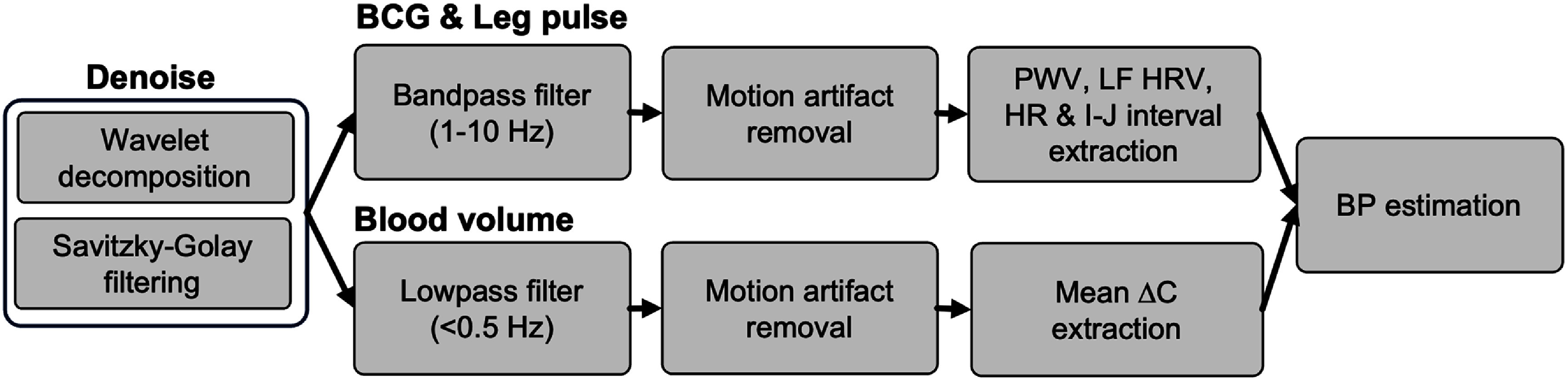
Flowchart of signal processing and feature extraction for BP estimation.

All signal processing, BP estimation, and analysis were conducted in MATLAB. Signals from sensing pads and reference devices were time-synchronized by aligning the system timestamps. Raw signals were denoised using wavelet decomposition (db4, level 2) followed by Savitzky–Golay filtering (4th order, window length 15). The denoised signals were then bandpass filtered (1–10 Hz) to obtain BCG and lowpass filtered (<0.5 Hz) to isolate intrathoracic blood volume variations (figure 3).

Each sensing pad contained four SEC sensors to expand coverage. Motion artifacts, identified by low inter-sensor correlation and phase misalignment, were excluded. Capacitance changes >0.5 pF caused by body shifts were removed (figure S4). Respiratory motion (∼0.2 Hz), which can introduce slow fluctuations affecting BCG-based features, was suppressed using a 1–10 Hz bandpass filter. The filtered 1–10 Hz components were averaged across sensors to enhance BCG signal strength.

For intermittent BP analysis, physiological features were extracted from 40 s windows during oscillometric arm cuff inflation/deflation cycle (figure S5). For continuous BP analysis, features were extracted from 10 s sliding windows with 5 s steps (0–10 s, 5–15 s, 10–20 s,). Reference beat-to-beat BP values were averaged within each window to mitigate noise and non-physiological fluctuations.

Within each window, BCG J-peaks and dominant leg pulse peaks were identified by envelope function to enhance amplitude maxima, followed by peak detection with distance and prominence constraints to select physiologically valid peaks (figure 1(b)). From these peaks, heart rate (HR), low-frequency HR variability (LF HRV, 0.004–0.15 Hz) (Mejía-Mejía *et al* 2020), and BCG I–J interval (Kim *et al* 2018) were derived. Mean Δ*C* was calculated as the average of the low-frequency (<0.5 Hz) sensor signal near the heart within each window. PTT was calculated as the time delay between the BCG J-peak and dominant leg pulse peak via cross-correlation. Pulse wave velocity (PWV) was then calculated as the pad spacing divided by PTT. Using the J-peak as the proximal timepoint avoided variability from the pre-ejection period seen in current ECG-based methods (Gesche *et al* 2012, Kim *et al* 2015).

### 2.4. BP estimation model

BP was estimated using a feature-based neural network (figure 1(b)). Inputs included mean Δ*C*, PWV (derived from PTT), HR, LF HRV, BCG I–J interval, age, and a baseline cuff BP for calibration. Age was included as a demographic factor. Separate models were trained for systolic BP (SBP) and diastolic BP (DBP). Architectures consisted of three fully connected layers (16–8–4 neurons for intermittent, 128–32–16 for continuous) with ReLU activations and an output layer. The loss function combined mean squared error, gradient and monotonicity penalties, and L2 regularization to constrain model complexity and limit overfitting. Model performance was evaluated with 10-fold cross-validation. The dataset was randomly split into ten equal subsets, with each iteration using nine folds for training and one for testing. Folds were rotated so every sample served once as testing data, and model parameters were reinitialized each iteration to ensure independent predictions. Small differences in root mean squared error (RMSE) between training and testing (<3% across iterations) indicated minimal overfitting (figure S6).

### 2.5. Statistical analysis

For intermittent measurements, a total of 137 data points were collected from 30 subjects performing repeated two BP-altering maneuvers. All statistical analyses were performed using estimations obtained from cross-validation. System performance for BP estimation was evaluated using the Pearson correlation coefficient (*r*), mean error (ME), mean absolute error (MAE), and standard deviation of error (SD). Agreement between the estimated and reference BP values was further assessed using Bland–Altman analysis, with the 95% limits of agreement calculated as ME ± 1.96 × SD.

For continuous measurements, a total of 1454 data points were collected from 8 subjects. Performance metrics including *r*, ME, MAE, SD, and 95% limit of agreement were evaluated. In addition, wavelet coherence analysis was performed to evaluate temporal alignment and morphological similarity between the estimated and reference dynamic BP signals. Finally, to quantify the contribution of mean Δ*C* as a novel variable for PTT-based BP estimation, we compared model performance with and without its inclusion (table S1).

## 3. Results

### 3.1. Comparison with arm cuff (intermittent)

**Figure 4.**
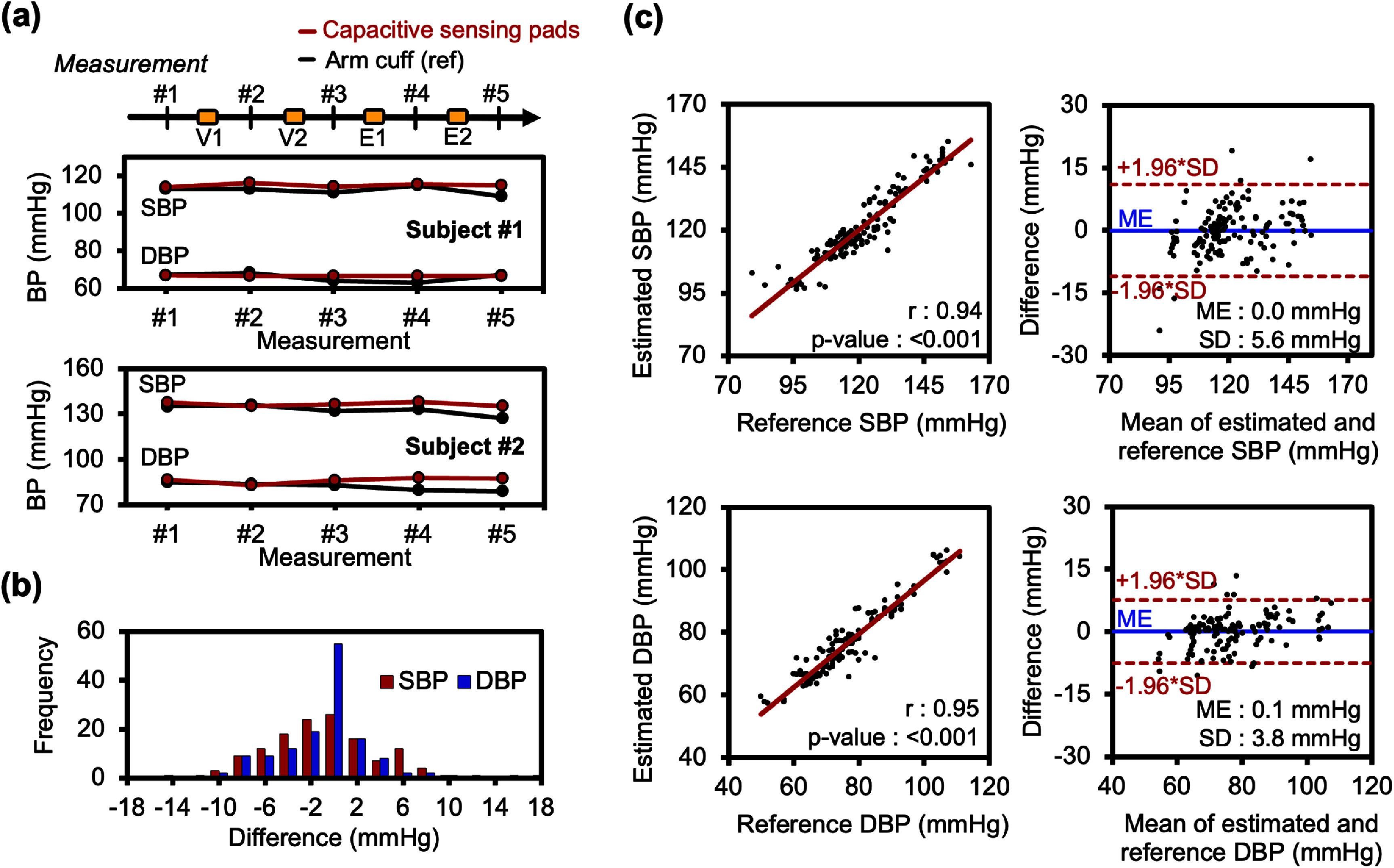
BP monitoring in a human study with an oscillometric arm-cuff reference (*N* = 30). (a) Representative results from two subjects performing Valsalva (V1, V2) and isometric handgrip (E1, E2) maneuvers, with 1 min rest periods for cuff measurements. (b) Histograms of SBP and DBP differences between capacitive pads and reference arm-cuff. Differences are normally distributed, with ∼95% within ±1.96 SD of the ME, indicating minimal subject-dependent variability. (c) Correlation and Bland–Altman analysis comparing BP estimation from the capacitive sensing pad with reference values. Pearson’s *r* = 0.94 for SBP and *r* = 0.95 for DBP (both *p* < 0.001). Estimation error (ME ± SD) is 0.0 ± 5.6 mmHg for SBP and 0.1 ± 3.8 mmHg for DBP; 95% limit of agreement is −11.0 to 11.0 mmHg (SBP) and −7.5 to 7.6 mmHg (DBP).

For validation against arm cuff intermittent measurements, a total of 137 BP data points were collected from 30 subjects. The capacitive sensing pads achieved an average signal-to-noise ratio (SNR) of 12.3. SNR was calculated using fast Fourier transform during baseline measurement, comparing capacitance power within physiological frequency bands (<0.5 Hz and 1–10 Hz) to power outside these bands. Representative BP data from the pads and the arm cuff are shown in figure 4(a).

Histograms of BP estimation errors (figure 4(b)) were approximately normally distributed, with ∼95% of errors within ±1.96 SD of the ME. Both SBP and DBP estimates closely matched the oscillometric reference, showing strong correlations with Pearson’s correlation coefficient *r* = 0.94 (*p* < 0.001) for SBP and *r* = 0.95 (*p* < 0.001) for DBP (figure 4(c)). The estimation error of BP was low (ME ± SD: 0.0 ± 5.6 mmHg for SBP, 0.1 ± 3.8 mmHg for DBP). Bland–Altman analysis yielded 95% limits of agreement (=ME ± 1.96 × SD) of −11.0–11.0 mmHg for SBP and −7.5–7.6 mmHg for DBP (figure 4(c)). These ME and SD values are comparable to ISO 81060-2:2019 standards for noninvasive BP monitors (ME ⩽ 5 mmHg, SD ⩽ 8 mmHg), though formal ISO compliance was not assessed (Association for the Advancement of Medical Instrumentation 2019). With baseline BP, the system showed strong correlation with the reference. After removing baseline BP offsets (Estimated − Baseline vs Reference − Baseline) (Mukkamala *et al* 2021), correlation was lower (*r* = 0.11 for SBP and *r* = 0.10 for DBP; Figure S7).

Feature importance was assessed using permutation analysis, where each feature was randomly shuffled to evaluate its impact on the RMSE of BP estimation (figure S8). The resulting RMSE increases were normalized to an importance score (scaled to 1) for relative comparison. Higher scores indicate greater importance. PWV was the main feature for SBP estimation, whereas mean Δ*C* was more influential for DBP estimation. To evaluate the contribution of mean Δ*C*, we compared it with the PTT model. Incorporating mean Δ*C* reduced the MAE by 17% and 37% for SBP and DBP, respectively (figure S9). Concordance analysis between each feature and reference BP further supported these findings (figure S10). For SBP, PWV showed a higher concordance rate (0.61) than mean Δ*C* (0.47), whereas for DBP, mean Δ*C* was higher (0.42) than PWV (0.40).

### 3.2. Comparison with finger cuff (continuous)

**Figure 5.**
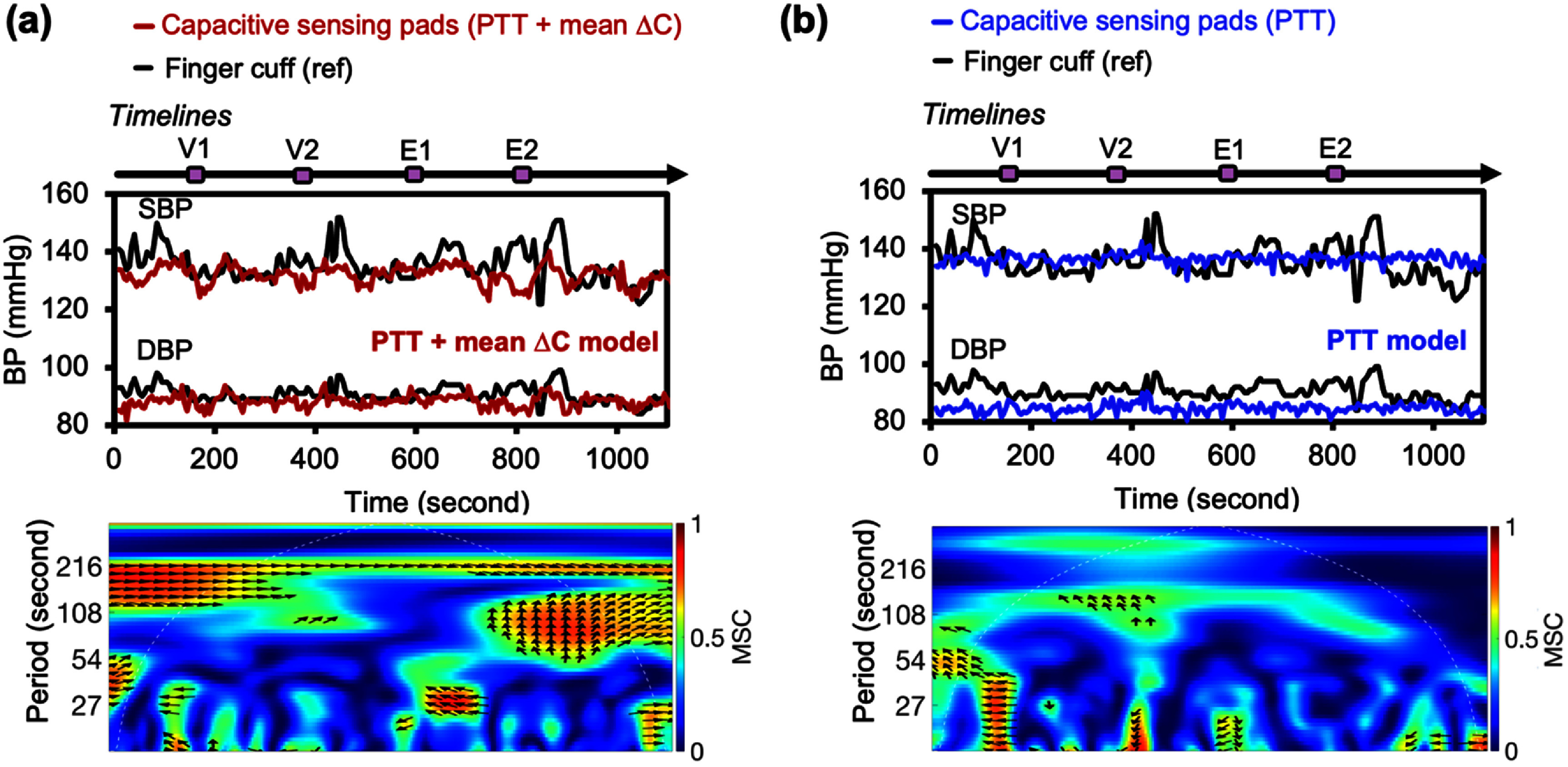
BP monitoring in a human study using a finger cuff as reference (*N* = 8). (a) Representative BP estimation from one subject using (a) PTT + mean Δ*C* or (b) PTT models. BP variation maneuvers include Valsalva (V1, V2) and isometric handgrip (E1, E2). Wavelet coherence spectrograms of MBP between the pad-derived signal and finger cuff reference. The *x*-axis denotes time, *y*-axis indicates time window size, and color scale shows MSC, with higher MSC reflecting stronger coherence. Black arrows indicate phase difference (rightward: in-phase; leftward: anti-phase). White dashed lines outline statistically reliable regions.

For validation against finger cuff continuous measurements, a total of 1454 BP data points were collected from 8 subjects (figure S11). Representative BP data from the capacitive sensing pads and the finger cuff are shown in figure 5. Consistent with intermittent results, the PTT + mean Δ*C* model (figure 5(a)) more accurately tracked dynamic BP compared to the PTT model (figure 5(b)).

Mean BP (MBP) was computed as MBP = DBP + (SBP − DBP)/3, and wavelet coherence analysis was performed between pad-derived and reference MBP (figure 5(a)). Magnitude-squared coherence (MSC) quantified signal similarity within each time window, with higher MSC values indicating higher coherence between signals. MSC remained high throughout monitoring, with an average maximum MSC of 0.71 (figure 5(a)). This indicates effective tracking of BP dynamics, representing a 34% improvement over the PTT model (figure 5(b); average maximum MSC = 0.53).

**Figure 6.**
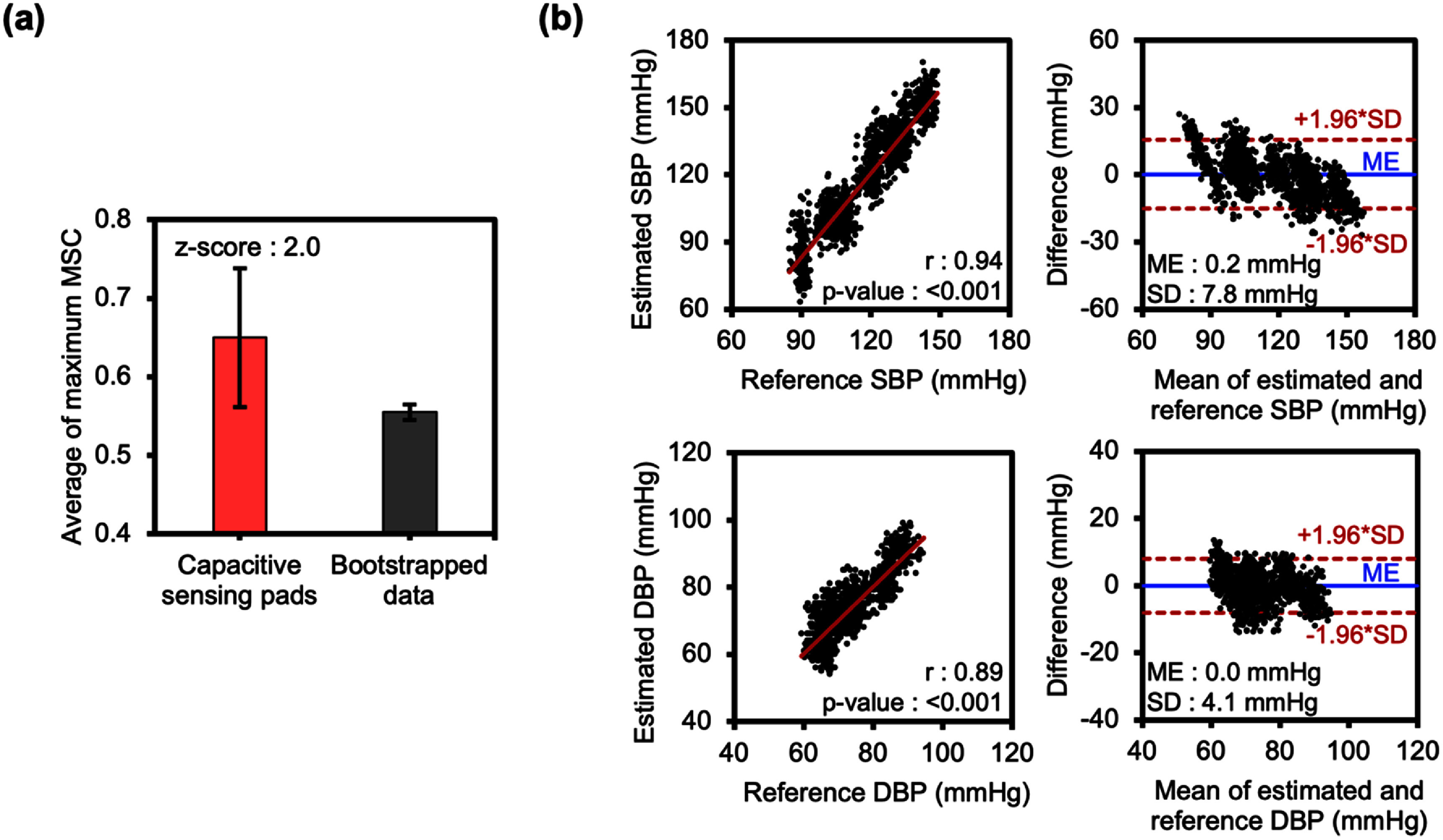
Evaluation of continuous BP monitoring in a human study using a finger cuff as reference (*N* = 8). (a) Comparison of average maximum MSC with the reference between bootstrapped data and pad-derived MBP. A *z*-score of 2.0, exceeding the 95% confidence threshold (1.96), confirms the statistical significance of the higher MSC in pad-derived data. (b) Correlation and Bland–Altman analysis comparing pad-derived BP with reference values. Pearson’s *r* = 0.93 for SBP and *r* = 0.89 for DBP (both *p* < 0.001). Estimation error (ME ± SD) is 0.2 ± 7.8 mmHg (SBP), 0.0 ± 4.1 mmHg (DBP); 95% limits of agreement is −15.1 to 15.6 mmHg (SBP), −8.0 to 8.0 mmHg (DBP).

To determine whether the high MSC occurred by chance, we applied bootstrapping by randomly shuffling the pad-derived MBP 100 times. Wavelet coherence analysis was then performed between (1) pad-derived MBP and the reference, and (2) bootstrapped data and the reference (figure S12). Comparing the average maximum MSC values across the session, pad-derived MBP showed significantly higher coherence with the reference (figure 6(a)). A *z*-score of 2.0, exceeding the 95% confidence threshold of 1.96, confirmed this similarity as statistically significant. These results demonstrated the capacitive pads’ ability to track dynamic BP trends.

Capacitive sensing pads also demonstrated strong correlation with finger cuff reference, with *r* = 0.93 (*p* < 0.001) for SBP and *r* = 0.89 (*p* < 0.001) for DBP (figure 6(b)). The estimated BP also had low error (ME ± SD: 0.2 ± 7.8 mmHg for SBP, 0.0 ± 4.1 mmHg for DBP). Bland–Altman analysis yielded 95% limits of agreement of −15.1–15.6 mmHg for SBP and −8.0–8.0 mmHg for DBP (figure 6(b)). Correlation decreased to *r* = 0.23 for SBP and *r* = 0.47 for DBP after removing baseline BP offsets (figure S13). Feature importance analysis (figure S14) identified mean Δ*C* as the key predictor for continuous SBP and DBP estimation. Incorporating mean Δ*C* alongside PTT (figure S15) reduced MAE by 2% for SBP and 26% for DBP.

## 4. Discussion

Incorporating mean Δ*C* into the PTT-based model significantly improved accuracy, particularly during continuous measurements with BP surges. By capturing intrathoracic blood volume changes, the model reflected rapid hemodynamic responses, including respiratory phase and global stroke volume fluctuations, which PTT alone may miss. Because MBP is physiologically proportional to cardiac output multiplied by vascular resistance (Yu *et al* 2020), mean Δ*C* provided additional information on cardiac output by reflecting blood volume variations that PTT alone did not capture. The improvement was more pronounced for DBP, likely because PTT derived from the leg systolic wave was less accurate for diastolic pressure (Ding and Zhang 2019). Furthermore, all physiological features showed concordance rates above 0.4 with reference BP (figure S10), allowing the machine learning model to exploit complementary and nonlinear interactions for accurate BP estimation.

The MAE of BP estimation did not differ significantly between male and female subjects (SBP: 3.4 ± 1.4 mmHg for males vs 5.1 ± 2.8 mmHg for females; DBP: 2.6 ± 1.6 mmHg for males vs 2.9 ± 1.0 mmHg for females). Using the Mann–Whitney *U* test, both comparisons yielded *p* > 0.05, indicating consistent model performance across sexes.

Benchmarking against prior systems (table S2) (Radha *et al* 2019, Yousefian *et al* 2019, Huang *et al* 2024, Bothe *et al* 2025), the capacitive sensing pads showed superior performance, with higher *r* and lower MAE and SD. Previous approaches relying solely on PTT or blood volume measures typically reported *r* < 0.85, MAE > 7.5 mmHg (SBP), >5.0 mmHg (DBP), and SD > 9.5 mmHg (SBP), >3.9 mmHg (DBP). PTT is sensitive to vascular tone, which can decouple it from BP. Intrathoracic (e.g., impedance cardiography) and peripheral (e.g., PPG) blood volume reflect cardiac output or local vascular changes but may relate nonlinearly to BP due to compensatory vascular responses. Combining both measures enables simultaneous tracking of arterial compliance and stroke volume, improving BP sensitivity.

The capacitive pads also demonstrated comparable performance to a pad-based optical fiber system to detect BCG waveforms for BP estimation using machine learning (Huang *et al* 2024). The fiber-optic system achieved low error during daytime testing, with ME ± SD of 0.1 ± 7.0 mmHg for SBP and 0.1 ± 3.5 mmHg for DBP. However, its measurement was limited to post-maneuver readings, missing dynamic BP changes during maneuvers. When tested for nocturnal BP, where substantial BP fluctuations occurred, the error increased to −0.3 ± 9.4 mmHg for SBP and −0.2 ± 4.8 mmHg for DBP.

With baseline BP calibration, the capacitive pads system showed strong correlation with the reference. Removing baseline offsets reduced correlation (Figures S7 and S12), showing the need for one-time calibration for cross-subject generalization. Nevertheless, without calibration, the PTT + mean Δ*C* model showed higher correlation with the reference than the PTT model for both intermittent (SBP: 0.11 vs 0.06; DBP: 0.10 vs −0.02) and continuous measurements (SBP: 0.23 vs −0.16; DBP: 0.47 vs 0.46), emphasizing mean Δ*C*’s value in tracking dynamic changes.

Capacitive sensing pads demonstrated potential for unobtrusive, continuous BP monitoring during supine sleep or rest. This approach could be valuable in acute or postoperative recovery settings, where patients are typically supine and still. In home settings, however, subjects may shift position, which can compromise signal quality and disrupt the fidelity of PTT. To address this, pads with larger and denser array of SEC sensors can be deployed. This configuration allows adaptive selection of the sensor beneath the chest and leg to maintain effective sensor–body contact and ensure accurate BP estimation.

Although current clinical guidelines recommend measuring BP in seated and supine position with the arm at heart level (Pickering *et al* 2005), subjects may change posture during sleep. In lateral positions, reduced contact with key arterial sites compromises the detection of intrathoracic blood volume, leg pulse, and PTT. Future work should focus on developing robust solutions for side-lying postures, such as leveraging the BCG morphology or identifying novel compensatory biomarkers. Finally, while our system successfully tracked dynamic BP changes, the absence of longitudinal data prevented a full assessment of calibration stability. Future studies should therefore prioritize improving nocturnal BP accuracy and extending calibration intervals to enhance long-term reliability.

## 5. Conclusion

We evaluated a capacitive sensing pads system for unobtrusive, continuous BP monitoring in supine subjects. The pads integrated ultra-sensitive SEC sensors made from carbon nanotube composites. Intrathoracic blood volume changes and BCG were captured from back of the chest, while pulse-induced vibrations were detected under the leg to derive PTT. A neural network incorporated these physiological features, including PWV and intrathoracic blood volume changes, to estimate BP. In human trials (*N* = 30) with oscillometric arm-cuff reference, the system showed strong correlation (SBP: *r* = 0.94; DBP: *r* = 0.95) with low error (ME ± SD: 0.0 ± 5.6 mmHg for SBP, 0.1 ± 3.8 mmHg for DBP). In a subset with continuous finger-cuff reference (*N* = 8), performance remained robust (SBP: *r* = 0.94; DBP: *r* = 0.89) with relatively low error (ME ± SD: 0.2 ± 7.8 mmHg for SBP, 0.0 ± 4.1 mmHg for DBP). Wavelet coherence analysis confirmed high coherence with reference, highlighting reliable BP tracking. These results demonstrate that integrating intrathoracic blood volume with PTT enhances accuracy and supports comfortable, continuous BP monitoring.

## Data Availability

The data that support the findings of this study are available upon reasonable request from the authors. Single electrode capacitance available at https://doi.org/10.1088/1361-6579/ae575a/data1.
